# 5 –year complex clinical and histopathological follow-up of a case of early gastric carcinoma (signet ring cells type)

**Published:** 2016

**Authors:** D Serban, C Branescu, C Savlovschi, G Simion, A Mihai, A El-Khatib, C Tudor, A Nica, G Vancea, M Ghelase, AM Dascalu

**Affiliations:** * ”Carol Davila” University of Medicine and Pharmacy, Bucharest, Romania; ** IVth Upper Digestive Surgery Clinic, University Emergency Hospital, Bucharest, Romania; *** University of Medicine and Pharmacy, Craiova, Romania

**Keywords:** early gastric carcinoma, multifocal, signet ring cell, surgery, histopathology

## Abstract

The paper presents the case of a male patient, hospitalized for acute abdomen due to perforated callous ulcer. Though the clinical appearance suggested a benign pathology, the histopathological exam of the resection piece showed multicentric early gastric carcinoma, signet ring cell type. At the patient’s request, total gastrectomy was not performed, a conservative solution being chosen instead. Superior digestive endoscopy with biopsy and oncological dispensarization was performed one month after surgery, then at every 6 months. After 2 years of benign results, the histopathological exam revealed the presence of malign singlet ring cells in the bioptic specimen. Respecting the patient’s option of preserving a good quality of life, subtotal gastrectomy with Pean type gastroenteroanastomosis was performed followed by postoperatory chemotherapy. Endoscopic and oncological follow-up were performed at every six months for another 3 years (up to present), and the evolution was favorable with no local or metastatic recurrence.

Histopathological examination was of great help in the surgical management of this case, allowing a fortunate early diagnosis, a conservative surgical approach, and the preserving of a good quality of life.

## Introduction

The management of early multifocal gastric carcinoma is challenging for the clinical practice. Aggressive surgical approach offers not only an oncological radical treatment but also an important impairment of the quality of life. Partial gastric resection, combined with strict postoperatory follow-up by endoscopy with biopsy, and histopathological exam are considered an efficient alternative by many authors [**1**-**6**].

## Case presentation

The paper presents the case of a 51-year-old male patient, hospitalized due to surgical acute abdomen, for which emergency surgery was mandatory. Anamnesis revealed a 30-years history of chronic gastritis, with inconstant medical and igieno-dietetic treatment, chronic consumption of alcohol, a previous surgery for a T3-T4 benign neurinoma, congenital left renal agenesia, and right renal ptosis. No family history of cancer was reported.

After laparotomy, a 5 cm callous gastric ulcer, with a central perforation at the medio-gastric level, on the greater curvature, with a macroscopic benign aspect, no pathological modifications of the adjacent lymph nodes, was found. A large excision was decided regarding the healthy (normal) gastric tissue, and the resulting pieces were sent to the pathological anatomy laboratory. Postoperatory evolution was rapidly favorable under antisecretory treatment, with the reinitiating of oral food intake after 4 days. 

The operatory resection piece of 5/2/1 cm was sent to the histopathological examination. 4 fragments were taken: 2 from the ulcerous area, 1 adjacent to the ulcer and 1 from the margins. Routine hematoxylin and eosin stain was performed. The microscopic examination revealed a gastric wall with corporal and antral mucosa with an ulceration of 0.8 cm extending deep into the second half of the muscularis propria. 

**Fig. 1:**
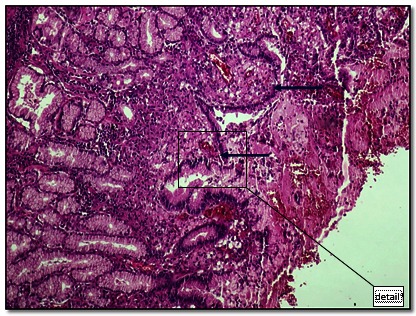
Patchy signet ring cells (arrows) without gland formation may be seen under the surface gastric epithelium, sustaining the diagnosis of early signet ring cell gastric carcinoma (see detail **Fig. 2**)

**Fig. 2:**
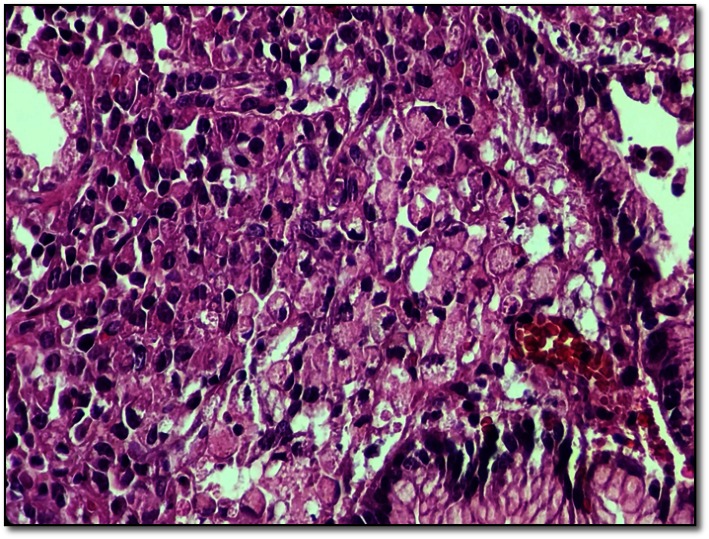
(detail) Patchy signet ring cells without gland formation may be seen under the surface gastric epithelium

At the continuity solution level of the ulcer, the histological modifications were of inflammation, with a predominant acute element towards the gastric lumen and predominant chronic limpho-monocitary population towards muscularis and serous layers. Adjacent to the ulcerous area, at the antrocorporeal level, limphoplasmocitary elements and malign signet ring cells were found at the level of the mucosa, mucosal glands and lamina propria, but spearing the muscularis mucosa and the submucosal layer. The resection margins (the gastric fundic and antral walls) were normal. The histopathological diagnosis was of multicentric early gastric carcinoma signet ring cell type, G3 (**Fig. 1**,**2**).

The early diagnosis in a case of signet ring cell gastric carcinoma is extremely rare and it was a histopathological discovery that was possible in the presented case due to the coexistence with a perforated gastric ulcer. The significance of the oncological limit resection in the case of a multicentric carcinoma loses its consistency, because small-undiagnosed foci may still coexist in different parts of the stomach. 2 treatment options were considered and explained to the patients: the radical one - total gastrectomy (and splenectomy) and a more conservative one – endoscopic and oncological follow-up. The patient’s decision was for the second alternative. He underwent a strict follow up program, by superior digestive endoscopy and oncological consult one month after surgery, then twice a year, at every 6 months. Every time, biopsies were taken and sent to the histopathological exam, revealing benign chronic inflammatory and regenerative elements in the gastric mucosa layer and stromal fibrosis.

The general evolution was good; the patient gained 4 kg in weight and succeeded in carrying on his normal daily activity. 2 years post surgery, at a routine endoscopic control, a suspect discolored area was observed in the distal part of the stomach. Biopsy of mucosa and submucosa was taken and malignant signet ring cells were evidenced on the hematoxylin-eosin stained tissue fragments. The patient chose again the least aggressive surgery, preserving a good quality of life. A subtotal gastrectomy with gastrojejunal Pean type anastomosis was performed. 

**Fig. 3:**
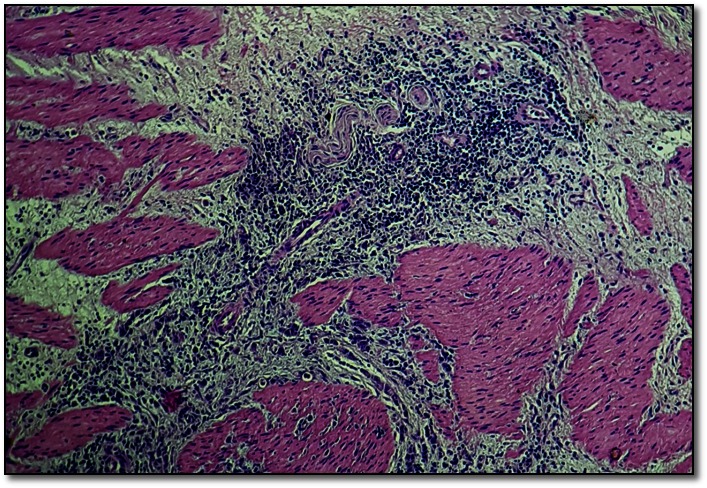
Atypical cells infiltrated between muscle fibers in the muscularis propria of the stomach

**Fig. 4:**
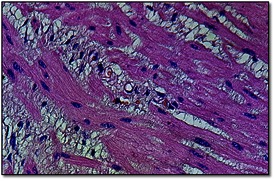
(detail) Atypical single signet ring cells infiltrated between muscle fibers into muscularis propria of the stomach

The anatomopathological exam revealed a recurrence of the signet ring cell carcinoma, with an invasion in the muscularis propria of the stomach (T2), localized in the antropyloric region. Atypical cells infiltrated between the smooth muscle fibers, with moderate inflammatory and desmoplastic stromal reaction (**Fig. 3**,**4**).

The postoperative evolution was good, with a reinitiating of oral food intake 6 days postoperatory. During the oncologic consult, 3 sessions of chemotherapy were decided, which the patient agreed with. The follow-up endoscopic and oncologic control was performed at every six months for the next 3 years (up to present). Local and general evolution was good, with no further recurrences.

## Discussions and conclusions

Signet ring cell gastric carcinoma is a special type of gastric malignancy, characterized by its diffuse, little cohesive properties, its low-grade differentiation, and sometimes-multifocal expression, being often associated with a poor survival prognosis. On the other hand, recent studies showed that this poor prognosis is not due to the histopathological feature itself, but to late diagnostic stage compared with adenocarcinoma. Sakuma and col. evidenced a favorable prognosis and a lower rate of lymph node metastasis in early signet ring cell carcinoma (SRC) compared to the early non-signet ring cell carcinoma, suggesting that the patients with early gastric carcinoma with SRC could be candidates for less invasive surgeries for an improved quality of life [**5**,**8**].

Another aspect to take into account in the presented case was the histologic diagnosis of multicentricity of the malignant foci. In a review of literature, it was stated that multicentricity might affect 3 to 22% of gastric cancers. Most of the SRC are located in the 2/ 3 distal of the stomach and antral mucosa, though the presence of malignancy in the 1/ 3 upper part could not be ignored [**2**,**5**,**8**]. Total gastrectomy is classically considered the radical therapeutic solution, but recent studies showed that there is no significant different 5-year survival rate for the patient with early multifocal gastric cancer who underwent subtotal gastrectomy in comparison with the early unifocal gastric cancer (90% vs. 92%), with the condition of a strict endoscopic and histopathologic postoperatory follow-up [**3**,**4**,**6**,**7**]. Synchronous multifocality of early gastric carcinoma does not increase the risk of lymph node metastasis compared with solitary early gastric carcinoma. Therefore, even the endoscopic treatment can be planned when major and minor lesions are predicted to represent mucosal cancer without lymphovascular invasion [**6**]. 

On the other hand, the reduction of the surgical extent and trauma under the premise of radical resection improves the quality of life of patients with gastric cancer [**4**]. In our particular case, together with the complications related to the esojejunal anastomosis, the risk of post splenectomy thrombocytosis and permanent antiaggregant therapy on the renal function and the demand of the patient for the least aggressive therapy had to be taken into account.

The significant rate of recurrence after partial gastrectomy justified follow-up by endoscopy, cytology, biopsy with histopathological exam. Even subtle endoscopic findings should be considered of potential risk and biopsied in order to locate gastric cancer early enough for the minimally invasive curative treatment to be feasible. The histopathological examination was of great help in the surgical management of this case, allowing a fortunate early diagnosis, a conservative surgical approach, and the preserving of a good quality of life.

**Disclosures**

None.

**Conflict of interests**

None.
